# Quantitative Behavioral Analysis and Qualitative Classification of Attachment Styles in Domestic Dogs: Are Dogs with a Secure and an Insecure-Avoidant Attachment Different?

**DOI:** 10.3390/ani11010014

**Published:** 2020-12-23

**Authors:** Giacomo Riggio, Angelo Gazzano, Borbála Zsilák, Beatrice Carlone, Chiara Mariti

**Affiliations:** 1 Department of Veterinary Sciences, University of Pisa, 56124 Pisa, Italy; angelo.gazzano@unipi.it (A.G.); beatricecarlone@gmail.com (B.C.); chiara.mariti@unipi.it (C.M.); 2 Department of Ethology, Institute of Biology, Eötvös Loránd University, 1117 Budapest, Hungary; zsilak.borbala@gmail.com

**Keywords:** attachment, strange situation procedure, proximity seeking, dog, dog-owner attachment, avoidant, secure, attachment style, insecure attachment, bond

## Abstract

**Simple Summary:**

Previous limited literature suggest that dogs present the same attachment styles as those observed in children towards their caregivers, namely secure, insecure-avoidant, insecure-ambivalent, and disorganized. However, such classification for dogs has never been corroborated by the simultaneous analysis of quantitative measures of behavior recorded during the Strange Situation Procedure (SSP). Since several modified SSPs have been used to investigate dog-to-owner attachment, in this study, two different groups of dog-owner dyads underwent two different versions of the SSP. Dogs were classified based on their attachment pattern toward the owner. For this purpose, we developed a new adaptation of the classification used for human infants. In addition, dogs’ behavior was recorded throughout the test. Behavioral differences between dogs classified as secure and avoidant were investigated. Results suggest that the classification used was effective at identifying secure and avoidant attachment styles in dogs. Like in children, behavioral differences between secure and avoidant dogs were particularly evident as the test progressed. While secure dogs showed an increase in proximity/contact seeking behaviors toward their owners, avoidant dogs did not. Larger samples may be needed in future studies to support these findings and to be able to include also ambivalent and disorganized dogs.

**Abstract:**

Since several modified Strange Situation Procedures (SSP) have been used to investigate dog-to-owner attachment, in this study two different samples of dog-owner dyads underwent two modified versions of the SSP. Dogs’ attachment style to the owner was assessed based on a novel adaptation of the attachment pattern classification used for infant-caregivers. Dogs’ behavioral data were collected using continuous sampling and, in the second protocol, also with a scoring system for greeting and social play. In both studies, secure and avoidant dogs’ behavior was compared using the Mann Whitney test, while differences within each group across episodes were analyzed using the Wilcoxon paired sample test. The classification seemed to be effective at identifying both avoidant and secure attachment patterns in dogs. As expected, differences in key attachment behaviors, such as proximity/contact seeking toward the caregiver, between secure and avoidant dogs were more evident in the final episodes of the test. Differently from secure dogs, avoidant dogs did not show an increase in proximity/contact seeking behavior with the caregiver in any of the procedures. Further studies with larger samples are needed to support the effectiveness of this classification and investigate on the remaining attachment styles.

## 1. Introduction

The attachment bond is a particular kind of affectional bond characterized by four essential elements: (1) contact maintenance that is maintaining physical contact and proximity with the attachment figure; (2) searching response (or protest at separation) when involuntarily separated from the attachment figure; (3) secure base effect, i.e., the attachment figure represents a base from which to explore the environment; (4) safe haven effect, i.e., the attachment figure provides a sense of safety in times of threat or distress [1,2,3,4,5]. The attachment bond is typical, although not exclusive, of the infant-parent relationship [6,7]. The Strange Situation Procedure (SSP) is a laboratory test that addresses the degree to which each of the essential elements of the child-caregiver attachment bond manifest themselves during partly standardized dynamics of interactions. Since its first implementation in the 1960s, the SSP is widely recognized as the golden standard procedure to assess attachment patterns of children toward their caregivers [8].

More recently, due to the growing interest in the field of anthrozoology, the SSP has been used to investigate the attachment bond between dogs and their owners. Topal et al. [9] were the first researchers using SSP to suggest that dogs show behaviors indicative of attachment toward their caregivers. They also provided statistical evidence that dog attachment behavior during the SSP could be divided into different categories, although they would not match the attachment patterns found in human infants. Since then, many researchers developed their own adaptation of the original SSP in order to test specific hypotheses about the attachment bond in dog-owner dyads and simultaneously avoid methodological issues arisen by the use of SSP on a different species than that for which it was originally developed [10,11,12,13]. Despite using different protocols, ethograms, sampling methods, statistical analysis, or even different laboratory procedures than the SSP, the great majority of these studies reached the conclusion that dog-owner relationship shares the same fundamental characteristics of the child-caregiver attachment bond (contact maintenance: [11,12,13]; separation distress: [11,14,15,16]; secure base effect: [11,12,17]; safe haven effect: [18]).

Most studies that used the SSP to investigate dog-owner attachment relied on the assessment of quantitative measures (i.e., frequency and duration) of dogs’ relevant behaviors. This is because quantitative measures allows for greater coding standardization and may represent the most useful approach to describe normative trends of behavior across episodes [19]. However, such measures have proved less useful in identifying individual differences in infant behavior in the SSP, as they make it more difficult to retain the qualitative aspects of the interactions that are at the bottom of pattern classification [19,20]. Recently, two pioneering studies by Schöberl et al. [21] and Solomon et al. [20] have adopted a qualitative approach to the study of dog-caregiver attachment bond by developing a dog-adapted version of the four-style classification used for human infants [22,23]. Schöberl et al. [21] correlated their classification to physiological parameters of stress during the SSP; they found a significantly lower cortisol reactivity in dogs classified as securely attached compared to those classified as insecurely attached. For statistical reasons, they did not perform analysis to differentiate between avoidant, ambivalent and disorganized subjects. Similarly, Solomon et al. [20], which used the same sample and classification procedure as Schöberl et al. [21], found a negative correlation of insecure classification to the caregiver’s reassuring presence during a “threatening stranger” test, as well as to the owner reported dog’s personality trait “active/excitable” on the Monash Canine Personality Questionnaire-Revised (MCPQ-R). Overall, the results of these studies suggest that dog and human attachment systems are organized over similar constructs, and that the style classification used for human infants may as well apply to dogs. Nevertheless, as Solomon et al. [20] pointed out in their manuscript, there is need for future research to confirm the suitability of infant attachment classification for dogs.

For instance, to date, there are no published studies that directly compare dog style classification to behavioral measures observed in dogs during the SSP [21], which has been an important step toward the validation of the attachment classification system in human infants [19,24].

Furthermore, in both Schöberl et al. [21] and Solomon et al.’s [20] studies, inter-observer agreement in relation to dog classification was, for their own admission, somewhat “elusive”. More precisely, observers could not reach an agreement on 22% of the cases, even after reciprocal confrontation. This issue highlights the need to create straightforward definitions of dog attachment patterns that guide the observers throughout the classification process and consequently increase their agreement.

The current study proposes a classification of the four attachment styles for dog-owner dyads that focuses on those critical general concepts that characterize each attachment pattern rather than on the description of precise behavioral sequences, with the ultimate goal of minimizing disagreement among observers and facilitate repeatability in future studies. Furthermore, we aimed to test the soundness of this classification by assessing differences in quantitative behavioral measures observed during the SSP among dogs classified with distinct attachment styles. Since several modified versions of the SSP have been used to investigate dog-to-owner attachment, we also aimed to assess whether the style classification we provided could be applied to two different SSP protocols.

## 2. Materials and Methods

### 2.1. Ethical Considerations

This study was carried out using videos collected for previous research on dog attachment published by Mariti et al. (for protocol I [25]; for protocol II [12]). As observational studies involving owned dogs, they did not require the approval by an ethical committee. In both cases, owner informed consent and authorization to video record were obtained before testing each dog. Before starting the tests, procedure was briefly described to the owner, but the specific goal of the studies was not disclosed.

### 2.2. Protocol I

#### 2.2.1. Subjects

Twenty-seven healthy, adult, pet dogs, were involved in the study. They were 62.9% females (12 intact and 5 spayed) and 37.1% males (8 intact and 2 castrated), their age ranging from 24 to 240 months (mean ± standard deviation age = 53.8 ± 40.5 months); twenty-one of them were pure-breed, while the remaining were mix-breed dogs. Demographic details for each subject are summarized in Table S1.

For all participating subjects, any behavioral and/or physical states or disorders (e.g., social or environmental phobias, aggressive behaviors toward people or conspecifics, estrus, pregnancy, evident painful conditions) that could affect the results of the test was ruled out through an interview carried out by a veterinarian.

Each dog was tested with his/her owner (74.1% women and 25.9% men). All owners were volunteers recruited through personal contacts. In case the dog lived in a multi-member family, the person participating was the one reported as the family member preferred by the dog. All dogs had been living in a home environment, alongside with the person they were tested with, for a minimum of six months.

The stranger was the only other person participating in the test. The stranger was always played by the same female researcher, who had never met the dog before. Her role was also that of guiding the owner throughout the entire test.

#### 2.2.2. Experimental Setting

The experimental environment was a relatively bare room, unfamiliar to the dogs, at the Department of Veterinary Sciences, University of Pisa (Italy). The room (4.50 × 4.30 m) was prepared to meet the description of the original ASST setting [3], as well as that given by later versions specifically modified for dogs [9,10,14]. The room was equipped with 2 chairs, 1 for the owner and 1 for the stranger; a water bowl; a table to lay the leash on; a single door around which a 1-m-radius semicircle had been drawn; and two video cameras (JVC Everio GZ-MG130E, Yokohama, Japan) to record the whole test. Because the videos were originally recorded with a different aim [25], toys were not present in the room. To avoid external noises, tests were conducted on weekends, when the building was not in use, for a total period of 4 months.

#### 2.2.3. Experimental Procedure

Before entering the room, the owners were instructed not to draw the dogs’ attention and to remain seated during the whole test, except when they had to leave or come back into the room, or when they had to comfort their dog in case of distress. The same attitude was requested to the stranger. The procedure presented the same number and order of episodes of the original Ainsworth’s SSP and it is described in detail below:

Introductory episode: Owner and dog entered the experimental room. The dog was unleashed and set free to explore the room, while the owner sat on a chair.

Episode 1.I: Owner and dog (2 min). The dog was left free to move and explore the room. 

Episode 2.I: Owner, stranger, and dog (2 min). The stranger entered the room. She could greet the dog if he/she sought attention. Then, she had to sit on a chair and ignore the dog although she could not move him/her away in case the dog approached her. 

Episode 3.I: Stranger and dog (2 min). The owner left the experimental room. He/she waited for his/her time to return, in another room 20 m away from the experimental setting. After the first minute, if the dog displayed evident signs of distress, the stranger could comfort him/her

Episode 4.I: Owner and dog (2 min). Owner returned to the experimental room. In the meanwhile, the stranger left. 

Episode 5.I: Dog alone (2 min). The owner left the room and the dog remained alone. If the dog displayed signs of distress for 60 s, the stranger could enter the room.

Episode 6.I: Stranger and dog (2 min). The stranger entered the room and could greet the dog, then she sat on a chair. If the dog displayed signs of distress, she could act as in episode 3. 

Episode 7.I: Owner and dog (2 min). Same procedure as in episode 4.

Instructions throughout the procedure were given by the stranger, who relied on a chronometer to keep time. After testing each dog, the experimental room’s floor was washed using a non-toxic, weakly scented disinfectant.

#### 2.2.4. Behavioral Data Collection

Data on the dogs’ behavior were collected through continuous sampling. Observations were made for the whole length of each episode. Only duration of the behaviors observed was recorded for the purpose of analysis. The ethogram (see Table 1) comprised behaviors described in the original study [25], as well as behaviors that had been observed and coded for the original study but had not been analyzed yet (i.e., avoidance of the owner, stress). The majority of the behaviors recorded in the original study were obtained from scientific literature [9,10,14,26,27], while others were introduced by the authors. Since the type of observation and coding made for the original study were suitable for the aim of the present investigation, they were not performed again. Observation and coding were performed by one researcher using the software Boris [28]. The coder was blind to the dogs’ classification.

#### 2.2.5. Classification of Dog Attachment Styles

A first attempt to classify dog attachment styles was made using Solomon et al. [20] descriptions (see Table 2). As a first step, 23 dogs were independently classified by two researchers. Inter-observer agreement was low, as only 15 out of 23 (65%) dogs matched their classification, even considering that authors provided a first and a second-choice option for classification. The un-matching cases were then discussed and re-evaluated by the two classifiers, but consensus was reached only for two additional cases, keeping a high disagreement of 26%. A possible issue related to inter-observer reliability was underlined also by the authors of that classification, who found that working together was needed to reduce a 22% of disagreement obtained working independently to a 11% obtained discussing disagreement and uncertainty [20].

Three of the researchers involved in the current study opened a discussion aimed at identifying the problems and possible solutions for such disagreement. Such discussion highlighted that most of the disagreement arose from two main points: a discrepancy in the classification of an individual dog based on different episodes (e.g., looking at descriptions provided by Solomon et al., [20], the same dog behaved as an insecure-ambivalent in episode 6 and as a secure in episode 7); a mix of behaviors displayed by a dog in a certain episode that descriptions reported as belonging to different styles (e.g., upon reunion with the caregiver, the dog seeks contact but soon lies down at a certain distance looking at the caregiver).

The decision was made to modify Solomon et al. [20] classification for the sample of dogs used in the current study; the modification was done based on classic classification of children attachment styles [19], thus reducing the importance of describing single behaviors and increasing the relevance of concepts such as the ability to reduce stress upon reunion. This process resulted in the style descriptions reported in Table 2.

The dogs were therefore re-classified based on the new descriptions (see Table 2). It was agreed that in case of uncertainty, prominence was given to episode 7, as the activation of the attachment behavioral system should make the style more evident [19]. With this classification system, a good inter-observer reliability between two researchers working independently on a preliminary sample of 20 dogs was reached (17 out of 20, i.e., 85.0%). An additional researcher and one of the researchers who participated in previous classification attempts were then involved in the assessment of the whole sample of the current study using the classification reported in Table 2. An agreement was found for 22 out of the 27 dogs observed (81.5%). The relative Cohen’s Kappa was 63.0%, considered a substantial agreement [29].

### 2.3. Protocol II

#### 2.3.1. Subjects

Forty healthy, adult, pet dogs, 42.5% females (*n* = 5 spayed, *n* = 12 intact) and 57.5% males (*n* = 3 neutered, *n* = 20 intact), participated in the study. Their age ranged from 16 to 50 months (mean ± standard deviation age = 41.4 ± 27.5 months). As for their breed, there were 32 pure-breed and eight mixed-breed dogs. Demographic details for each subject are summarized in Table S1.

Before the test, the dogs underwent a veterinary consultation to rule out the presence of behavioral disorders and/or physiological states (e.g., social or environmental phobias, aggressive behaviors toward people or conspecifics, estrus, pregnancy, evident painful conditions) that could have an effect on the results.

During the test, each dog was accompanied by his/her owner (20 women and 20 men), who were volunteers recruited through personal contacts. In case the dog lived in a multi-member family, the person participating was the one reported as the family member preferred by the dog. All dogs had been living in a home environment, with that person, for a minimum of 6 months.

The owner, the dog, and a stranger participated simultaneously in the experimental phase. The stranger was always played by the same female researcher, who had never met the dog before and guided the owner throughout the whole test. The female researcher was not the same as in protocol I.

#### 2.3.2. Experimental Setting

The experimental setting was the same as in protocol I. The only difference was the presence of toys (a Kong, a puppet, and a rope) within the experimental room. Also in this case, the tests were performed on weekends over a period of 4 months.

#### 2.3.3. Experimental Procedure

The procedure has been described in detail in Mariti et al. [12]; below only salient points of the modified SSP will be reported.

The entire procedure comprised a pre-experimental (to fill in a questionnaire and the Lexington Attachment to Pets Scale (LAPS) [30], whose results are not presented in this study) and an experimental phase, the latter divided into seven episodes plus an introductory episode. The participants were asked to remain seated during the whole procedure, except when they had to leave or come back into the room and during play sessions, as well as not to begin interactions with their dog except in those phases during which they had to stimulate the dog to play.

The experimental phase was divided into the following episodes:

Introductory episode: Owner, stranger, and dog entered the experimental room. The dog was unleashed and set free to explore the room, participants sat on the chairs.

Episode 1.II Owner stranger, and dog (3 min). At the end of the third minute, the owner left the room.

Episode 2.II: Stranger and dog (2 min plus time needed to stimulate the dog to the social play). In the first minute, the stranger had to ignore the dog, even if he/she was seeking attention. In the second minute, the stranger pulled out owner’s shoe from the plastic bag and put it on the empty chair. The stranger could only interact with the dog if he/she was seeking attention. At the end of the second minute, the stranger tried to stimulate the dog to play, with a maximum of three trials (1 for each toy in the room). As soon as the dog started playing, or at the end of the third trial, the stranger called the owner back into the experimental room.

Episode 3.II: Owner, stranger, and dog (2 min plus time needed to stimulate the dog to the social play). The owner came back into the room. In the first minute, the owner knocked on the door and stayed behind it for 10 s. At the end of the 10 s, the owner entered the room and stayed for 50 s within 1 m of the door, to allow the dog to greet him/her. If the dog initiated interaction, the owner could greet and comfort the animal he/she would usually do at reunion. In the second minute, the owner sat down and took the shoe off the chair. At the end of the second minute, the owner tried to stimulate the dog to play, with a maximum of three trials (1 for each toy present in the room). As soon as the dog started playing, or at the end of the third trial, the stranger left the room.

Episode 4.II: Owner and dog (2 min plus time needed to stimulate the dog to the social play). Same as episode 2, but with roles inverted for owner and stranger.

Episode 5.II: Owner, stranger, and dog (2 min plus time needed to stimulate the dog to the social play). Same as episode 3, but with roles inverted for owner and stranger.

Episode 6.II: Dog alone (1 min). The dog was left alone in the room.

Episode 7.II: Owner, stranger, and dog (1 min). The owner and the stranger came back into the room. If the dog initiated interaction, the persons could greet the dog. The participants sat in the same chairs as before and made conversation.

Instructions during the test were announced by the stranger, who relied on a chronometer to keep time. When the stranger was outside the room, she gave instructions in a quiet voice. At the end of each test, the experimental room’s floor was washed using a non-toxic weakly scented disinfectant.

#### 2.3.4. Behavioral Data Collection

Quantitative behavioral data were collected using two methods: a continuous sampling observation and a scoring system.

As for the continuous sampling, behavioral observation focused on specific time frames within each episode. During episodes 3.II and 5.II observation lasted 50 s, starting when the person entered the room and ending when he/she walked to the chair; during episodes 2.II and 4.II the observation lasted 60 s, starting when the person’s shoe was placed on the chair and ending at the beginning of the play session; in episode 6.II the observation lasted 60 s, starting when both owner and stranger left the room; in episode 7.II the observation lasted 50 s, starting as soon as the owner sat on the chair.

The ethogram comprised behaviors used in the original study [12], as well as other previous studies on dog behavior [26,27,31,32,33]. Some behaviors were added by the authors for the specific purpose of the current study. Duration (in sec) and/or frequency (calculated as number of occurrences per minute) were recorded depending on the behavior observed (Table 3). Unlike protocol I, observation and coding performed for the original study were not suitable for the purpose of the current investigation, hence they were performed again. Observation and coding were performed by one researcher using the software Boris [28]. The coder was blind to the dogs’ classification.

As for data collected through scoring, dogs’ social play behavior [12] and greeting behavior [9] toward both the owner and the stranger were observed and scored as reported in Table 4. Greeting observation started when the person entered the room and lasted at maximum 10 sec; for its calculation, only behaviors initiated by the dog was taken into account. The final greeting score corresponded to the score of the very first action initiated by the dog (e.g., full approach = +2) and, in case the dog did another action within two seconds, the first was summed to the score of the second action (e.g., avoidance = −1; in this example, the greeting score was 2 − 1 = +1); its final value could range between −1 and +2. Social play observation started at the beginning of third minute of the corresponding episodes and ended as soon as the dog responded to the person’s play invitations or after 60 s if the dog could not be engaged.

The behavioral scoring system was applied only to protocol II because of some differences in protocol I that did not make it suitable for this type of assessment (e.g., in protocol I, the person entering the room immediately went to the chair, the greeting was very short and therefore and more easily altered by the person’s behavior; while in protocol II the person was asked to spend 50 s in front of the entrance door before moving to the chair, to have the opportunity to carefully observe the dog’s response to his/her arrival).

#### 2.3.5. Classification of Dog Attachment Styles

Dogs were classified using the attachment style definitions developed and used for the dogs in protocol I. In this case, inter-observer agreement was 82.5% (33 out of 40 dogs) before and 87.5% (*n* = 35) after reciprocal confrontation. The relative Cohen’s Kappa was 71.0%, considered a substantial agreement [29].

### 2.4. Statistical Analysis

#### 2.4.1. Statistics for Protocol I

For protocol I, possible behavioral differences between and within groups of dogs classified with distinct attachment styles were analyzed. Only groups with several dogs higher than 5 were retained for statistical analysis, namely dogs classified as secure and avoidant. One ambivalent and two unclassifiable dogs were excluded from further analysis.

The first step of the statistical analysis consisted of comparing the quantitative behavioral measures observed in the secure and the avoidant groups for episodes from 3.I to 7.I using a U-Mann Whitney test (multiple comparison corrections were performed using the Benjamini-Hochberg procedure). The statistical analysis was focused on episodes where the attachment system was activated (separations or reunions); episodes 1.I and 2.I were therefore excluded from the analysis.

The second step was a within-group comparison for both secure and avoidant groups. The comparisons for all behaviors observed were made based on the following rationale: episode 3.I (1st separation from the owner, stranger present) versus 4.I (1st separation from the stranger, owner present), 5.I (dog left alone) and 6.I (reunion with the stranger after 2nd separation, owner absent); episode 6.I versus episode 7.I (reunion with the owner after 2nd separation, stranger absent); episode 4.I (1st reunion with the owner, stranger absent) versus episode 7.I All behaviors analyzed and the corresponding episodes for which comparisons were made are summarized in Table S2.

The Wilcoxon paired sample test was used, applying the Bonferroni correction for multiple comparisons.

#### 2.4.2. Statistics for Protocol II

Also for protocol II, possible behavioral differences between and within groups of dogs classified with distinct attachment styles were analyzed. Only groups with several dogs higher than 5 was retained for statistical analysis, i.e., dogs classified as secure and avoidant. Two dogs classified as ambivalent were excluded from further analysis.

Behaviors collected through the continuous sampling observation, as well as those assessed through the scoring system were firstly analyzed comparing the secure and the avoidant groups for episodes from 2.II to 7.II using a U-Mann Whitney test (multiple comparison corrections were performed using the Benjamini-Hochberg procedure). Then, a within-group analysis for both secure and avoidant groups was performed based on the following rationale: for proximity seeking and looking at person, comparison was carried out for behaviors toward the stranger and the owner at the second reunion (both in episode 7.II), toward the stranger and the owner when all the three were present (episode 3.II versus 5.II), toward the owner during the first and second reunion (episode 3.II versus 7.II), and when only one person and the dog were present (episode 2.II versus 4.II); for behaviors related to protest at separation a comparison was carried out between the first and second separation from the owner (episode 2.II versus 6.II); for stress behaviors, a comparison was made between first separation from the owner and first separation from the stranger (episode 2.II vs. 4.II), as well as between first and second separation from the owner (episode 2.II vs. 6.II); for greeting interruption a comparison was made for behaviors toward the stranger and the owner when all the three were present (episode 3.II versus 5.II), toward the owner during the first and second reunion (episode 3.II versus 7.II) and toward the stranger during the first and second reunion (episode 3.II vs. 5.II); for individual play and exploration comparison were carried out between first separation from the owner and first separation from the stranger (episode 2.II vs. 4.II), as well as between first and second separation from the owner (episode 2.II vs. 6.II).

Data collected through the scoring system were analyzed using the Wilcoxon test as follows. For the greeting scores at reunion, scores toward the owner were compared at the first and second reunion (episode 3.II versus 7.II), scores toward the stranger were compared at the first and second reunion (episode 5.II versus 7.II), scores toward owner and stranger were compared at the first reunion (episode 3.II versus 5.II) and at the second reunion (episode 7.II). For the social play, scores toward the stranger in the absence and presence of the owner (episode 2.II versus 5.II), scores toward the owner in the absence and presence of the stranger (episode 4.II versus 3.II), scores toward the stranger and the owner when all the three were present (episode 3.II versus 5.II) and when only one person and the dog were present (episode 2.II versus 4.II). All behaviors were analyzed and the corresponding episodes for which comparison were made are summarized in Table S2.

## 3. Results

### 3.1. Protocol I

According to the style classification used in this study the distribution of each style within the sample was the following: secure 18 (72%), avoidant 6 (24%), ambivalent 1 (4%), and unclassifiable 2 (8%).

Comparison of behaviors between avoidant and secure dogs revealed some significant differences. Proximity seeking behaviors toward both owner and stranger were always significantly greater in secure dogs compared with avoidant dogs. On the contrary, behaviors related to protest at separation were greater in avoidant dogs compared with secure dogs. All statistical tendencies with 0.05 ≤ *p* < 0.1 and statistically significant results with *p* < 0.05 are summarized in Table 5.

Several differences were found comparing secure dogs’ behavior, as well as avoidant dogs’ behavior, across episodes. Most importantly, secure dogs showed an increase in proximity/contact seeking behaviors toward their owners as the test progressed, whereas avoidant dogs did not. Furthermore, while secure dogs constantly showed significantly greater protest at separation when the owner was absent compared with when the stranger was absent, avoidant dogs did so only during the first bout of separations. All statistically significant results and tendencies are reported in Table 6 and Table 7 for secure and avoidant subjects, respectively.

### 3.2. Protocol II

Based on the four-style classification scheme previously described, 32 (80%) out of the 40 dogs involved in protocol II were classified as secure, six (15%) as avoidant, two (5%) as ambivalent. None of the dogs was classified as disorganized or remained unclassified. Secure and avoidant dogs were retained for following results.

Comparison between secure and avoidant subjects’ behavior during the SSP also revealed some significant differences in all the analyzed episodes, except episode 5.II. For instance, proximity/contact seeking behaviors toward the owner were significantly greater in secure dogs in all reunion episodes. On the contrary, behaviors related to protest at separation were significantly greater in avoidant dogs when the stranger was absent, as well as when the dog was left alone. Again, all statistically significant differences and tendencies are reported in Table 8.

Moreover, significant differences in the behavior of dogs classified as secure and avoidant, were revealed across episodes. Secure dogs displayed greater proximity/contact seeking behaviors toward the owner than toward the stranger throughout the procedure. During the second reunion they showed fewer greeting interruptions. They also tended to play more with the owner than with stranger. Furthermore, they played more with the stranger when in the owner’s presence rather than in his/her absence. Importantly, avoidant dogs’ proximity/contact seeking behavior did not significantly differed neither between owner and stranger nor with the progression of the procedure. All significant results are summarized in Table 9 and Table 10 for secure and avoidant dogs, respectively.

**Figure 1:**
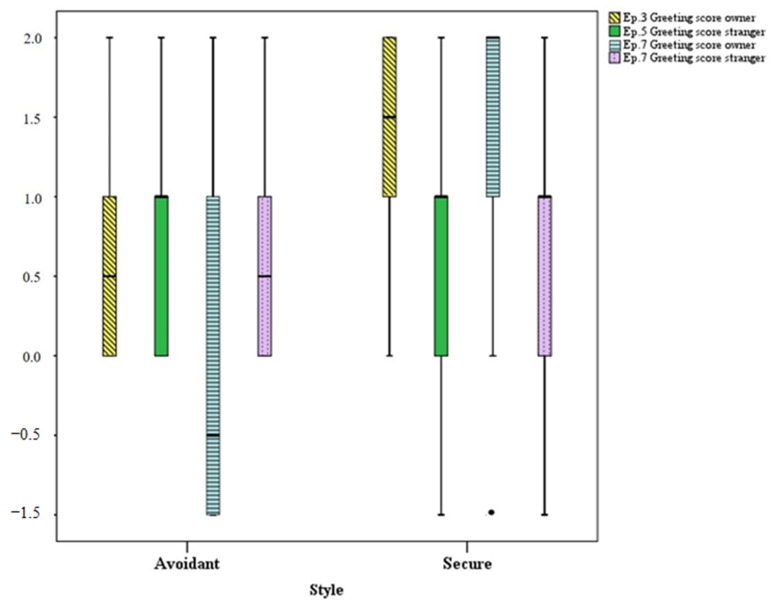
Greeting scores in avoidant (*n* = 6) and secure (*n* = 32) dogs in episode 3.II (toward the owner—yellow box), 5.II (toward the stranger—green box) and 7.II (toward both the owner—blue box and the stranger—purple box). For each box, the bottom and top horizontal lines represent the lowest and highest values, the lowest and top edge of the box represent the lower and upper quartile, the black horizontal line within box represents the median, and the dots represent the outliers.

**Figure 2:**
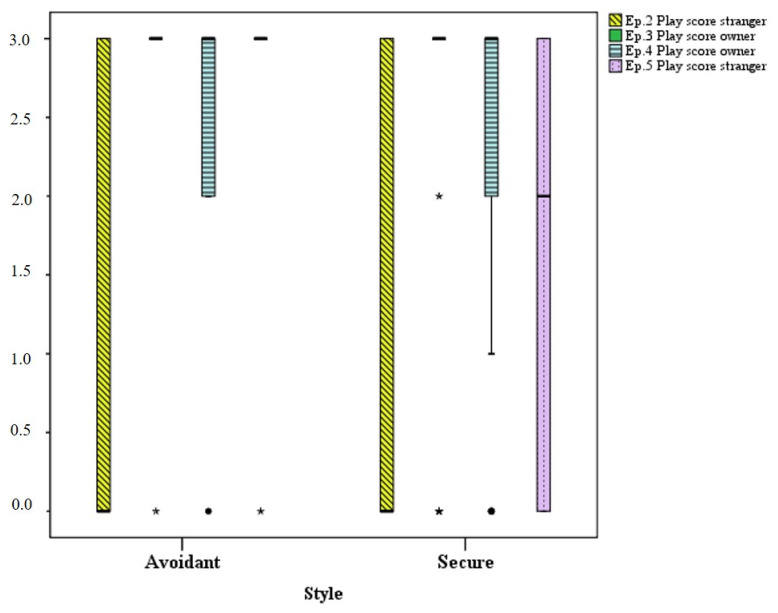
Social play scores in avoidant (*n* = 6) and secure (*n* = 32) dogs in episode 2.II (toward the stranger—yellow box), 3.II (toward the owner—green box), 4.II (toward the owner—blue box) and 5 (toward the stranger—purple box). For each box, the bottom and top horizontal lines represent the lowest and highest values, the lowest and top edge of the box represent the lower and upper quartile, the black horizontal line within box represents the median, and the dots and the stars represent the outliers. For those episodes where only the black horizontal line is visible, median, and lower and upper quartile values overlap.

Figure 1 and Figure 2 show, respectively, differences in scores for greeting and social play behaviors between both groups in the analyzed episodes. In Figure 1, avoidant dogs show a tendency to greet the owner less than the stranger during reunions, as well as a to decrease greeting behavior toward the owner in episode 7 (second reunion) compared to episode 3 (first reunion). On the contrary, secure dogs show significantly more greeting behavior toward the owner than toward the stranger in all reunion episodes, as well as a significant increase in greeting behavior toward the owner in episode 7 compared to episode 3.

In Figure 2, avoidant dogs do not show any difference in social play behavior during reunions with the owner (episode 3) and with the stranger (episode 5). On the contrary, secure dogs play with the owners significantly more than the stranger in all compared episodes (episode 2 vs. episode 4 and episode 3 vs. episode 5).

## 4. Discussion

The study of the attachment bond in dogs is traditionally carried out using the SSP, based on some similarities between the child-caregiver and the dog-caregiver bond. This approach has provided many relevant results since its first use [9] and highlighted that the theory formulated by Bowlby, and widened by Ainsworth and other colleagues, has many features in common with the bond linking dogs to their caregivers. However, it is also important to underline that different responses due to age [34] and species [12] are likely to exist, thus requiring a critical interpretation of the SSP results. This critical approach has been stressed when analyzing the possible intraspecific attachment in adult domestic dogs [27,35], but also the study of the interspecific attachment may benefit from taking into account the differences of examining a dog-caregiver rather than a child-caregiver bond [7]. The following discussion will be based, on the one hand, on highlighting the dog-child similarities observed in the SSP; and, on the other hand, on providing possible explanations for the differences observed.

This is the first study on dog-owner attachment to directly compare quantitative measures of dog behavior during the SSP with the qualitative classification of their attachment style. Such comparison represents an important step in order to assess the validity of the attachment style classification system in dogs [21]. Another aspect of novelty of the present study is that avoidant and ambivalent subjects were not grouped into a broader insecure category as previous studies did [20,21], but we excluded the few ambivalent dogs from the analysis and focused on the comparison between secure and avoidant individuals. This decision was made since ambivalent and avoidant dogs supposedly display markedly different behavioral patterns during the SSP. Hence, analyzing their behavior as a group may have masked relevant behavioral patterns from each style.

Overall, both protocols used in the current study seemed to be suitable to identifying the normative trends of behavior that we expected to characterize secure dogs. However, protocol I seemed to be the most suitable at revealing expected differences in secure dogs’ behavior across episodes in quantitative measures deriving from behavioral observations, while significant results in protocol II were mainly given by differences in social play and greeting scores.

As for the comparison of avoidant dogs’ behavior among the episodes of the SSP, we did not find as many significant differences as in the case of secure dogs. However, the low number of positive results is consistent with the low-keyed, inhibitory facet of the avoidant attachment style. As we expected, the avoidant subjects of this study did not increase their proximity/contact seeking behavior toward neither the caregiver nor the stranger, with the progression of the test; nor they intensified their manifestations of separation distress [19]. On the opposite, we may have expected these dogs to increase their avoidance and therefore decrease their proximity/contact seeking behavior toward the caregiver as the test progressed and their attachment style supposedly became more evident [19]. However, this did not seem to occur. Perhaps, the use of additional scoring categories, other than greeting and social play behaviors, may help show more clearly the dynamics of interaction of these subjects throughout the test.

In protocol I, avoidant dogs displayed more behaviors indicative of separation distress during the first separation from the owner rather than during the first separation from the stranger. It is interesting to note that during the second bout of separations such difference was not revealed. Again, repeated separations may be necessary in order for the specific attachment style to manifest fully. Avoidant subjects also explored more during owner’s rather than stranger’s presence in the first part of the procedure. Ainsworth [19] claimed that although exploration in the presence of the caregiver may be seen as a sign of the secure base effect, in the case of avoidant individuals it may be interpreted as a sign of displacement behavior. Although we are not stating that this is the case, because we did not discriminate true exploration from exploration as a displacement behavior in our behavioral observations, we cannot exclude that this may explain why avoidant subjects explored more in the presence of the owner rather than in his/her absence.

In protocol II, avoidant dogs appeared more distressed when they were left alone rather than when they were left in the presence of the stranger. A prerogative of avoidant subjects is that of showing little, if any, overt distress at separation; however, it is not rare for avoidant infants to show higher levels of stress when left alone compared to when they remain in presence of the stranger [19], and this could also occur in dogs.

This is the first study to include stress-related behaviors into the analysis of quantitative behavioral measures in the SSP. Results do not match our expectation that secure dogs would display more stress-related behaviors than avoidant dogs in the absence of the caregiver. However, in protocol I there seems to be a pattern of avoidant dogs vocalizing more, while secure dogs displaying more stress behaviors, in the presence of the owner. Among secure dogs, there also seems to be a pattern characterized by a significantly greater display of stress signals in the presence of the owner, as well as significantly longer times spent vocalizing in his/her absence. To be able to interpret these results in the context of the SSP and attachment styles, we believe that the necessary knowledge of the communicative function of different stress-related behaviors, the different contexts in which each of them may be displayed, as well as the relationship between the level of emotional distress and its phenotypic display, has not yet been achieved

The final classification used in this study was developed following the same procedure reported by Ainsworth [19] for infants and more recently by Solomon et al. [20] for dogs. However, contrary to the latter, we achieved a higher inter-observer agreement even before reciprocal confrontation. We believe that this may be due to the fact that our classification gives more weight to those key concepts that characterize the evolution of attachment behavioral patterns throughout the test rather than focusing on specific behaviors or behavioral sequences within each episode. For instance, for the secure style description we introduced the sentence “the reunion with the caregivers (their mere presence or, for other dogs, the proximity or the contact with them) is responsible for calming the dog in case he was distressed at separation”, while for the avoidant dog group we added the phrase “the dog does not show marked preference for the caregiver”.

This approach may have led to the different results in terms of distribution of attachment patterns. In accordance with the findings from previous research on dog attachment [20,36,37], the secure style was the most represented. However, in both our studies, the percentage of avoidant subjects was higher than that of ambivalent ones. In particular, ambivalent dogs in our sample were notably less numerous than those found in previous studies on dogs. For instance, Wanser and Udell [37] found 1% avoidant and 44% ambivalent in their sample of animal assisted activity trained dogs. In their study, Thielke et al. [38] classified within the shelter group 9.7% as avoidant and 51.6% as ambivalent, and within the foster group 4.8% as avoidant, 38.1% as ambivalent, and 4.8% as disorganized. Finally, Solomon et al. [20] classified 14% as insecure-ambivalent, 6% as insecure-avoidant, and 20% as disorganized.

Alongside with the type of classification used, dissimilarities in style distribution may be due to different methodological factors. Firstly, it is likely that some dogs’ previous experiences, such as being surrendered or rehomed- as in the case of shelter and foster dogs- or being trained to interact with people—as in the case of AAA dogs-, may affect the way they relate to humans or the way they behave during the SSP [37]. Moreover, when taking dogs’ previous experiences into account, one should consider that ambivalence may be displayed by dogs that have been taught to control their behavior in situations of high emotional arousal, as it may occur when greeting the owner upon reunion. This is because, most of the times, this type of training results in a behavioral inhibition rather than an actual reduction of the dog’s internal level of arousal, which may be particularly high when greeting occurs during stressful conditions, such as repeated separations in an unfamiliar environment. Nevertheless, in these cases, ambivalent behavior may not be related to the type of relationship with the owner, but rather be a consequence of the conflicting motivations behind that specific interaction. Secondly, in order to participate to any of our procedures, dogs did not have to present any behavioral disorders, which was not a specified requirement in Solomon et al. [20] recruitment process. This may be regarded as a major methodological difference, as behavioral disorders have been linked to insecure attachment styles in humans [39,40,41]. Thirdly, some authors [36,37,38,42,43] based their classification on a three-episode version of SSP named the Secure Base Test (SBT) that comprises one separation and one reunion episode, which as suggested by Ainsworth et al. [19], may not be distressing enough for the tested individual to display a recognizable attachment pattern. In fact, the original eight-episode SSP has been developed after extensive observation of infant-mother dyads in naturalistic environment; it is specifically designed to progressively increase the level of stress of the subjects tested and, consequently, to activate their attachment behavioral system. Therefore, as suggested by Solomon et al. [20], any changes made to the original protocol, although possibly useful to answer specific experimental questions, may alter relevant features of dog attachment patterns. Results from the present study seem to support such statement, especially with regard to the use of single separation procedures. In fact, in both our protocols, differences in proximity seeking behavior between secure and avoidant dogs are more evident upon the second reunion. It is plausible that the increased level of stress generated by the second separation activates the dog’s attachment behavior at a higher intensity, making the related pattern more distinguishable.

This study has some limitations. By excluding dogs with behavioral disorders, we may have altered the results in terms of relative percentage of each attachment style within our sample. Furthermore, the size of our sample was relatively small. Therefore, the distribution of attachment styles within our sample may not be representative of the entire dog population. In addition, due to the small number of dogs classified as ambivalent, we had to exclude them from statistical analysis. A greater number of experimental subjects may be necessary in future studies in order to draw a clearer picture of the attachment style distribution among dogs. Another limitation is that both protocols differed from the original Ainsworth’s SSP [2,3] in one or more aspects. This is because, as we previously mentioned, they were developed for different purposes than that of the present study. Furthermore, both protocols differed among each other in terms of ethogram and analyzed variables. Of course, this may affect the interpretation of some of our findings. Overall, considering the current results, we suggest that future attempts to classify dog-to-owner attachment style should rely on a protocol as similar as possible to the original SSP, in terms of episode number, episode order and use of scores of interactive behaviors that can quickly and more effectively measure some aspects directly related to the bond [19]. As for the behaviors displayed by people during the test, on one hand it is important to have a good degree of standardization, in order to allow dogs to initiate their search of proximity regardless of the owner behavior, as well as to compare quantitative data across episodes. On the other hand, since individual differences in attachment relationship are the result of repeated dyadic interactions, we suggest that future studies give higher prominence to the human part of the dyad, by introducing dedicated sections of interaction, possibly not or little standardized, within [44] or even outside of the SSP [20], allowing an analysis of the caregiver’s behavior and of the dog’s behavior in response to that.

## 5. Conclusions

This is the first study on dog-to-owner attachment to compare quantitative measures of dog behavior observed during the SSP with the holistic evaluation of their attachment style. It is also the first study to analyze avoidant dogs’ behavior separately from that of other insecurely attached subjects.

Two modified SSPs were used. Both protocols seemed to be suitable at revealing expected trends of behavior of secure dogs across the SSP, although expected differences in quantitative behavioral measures were more numerous in protocol I. As for avoidant dogs, although no positive results were found that allowed us to unambiguously determine the normative trend of behavior of this group, the low number of significant behavioral differences across episodes is consistent with the inhibitory aspect of the avoidant attachment style. Overall, expected behavioral patterns of both groups became more evident as the tests progressed.

We suggest that future studies aimed at assessing dog-to-owner attachment rely on SSPs as similar as possible to the original test and avoid shortened versions and/or structural modifications. Furthermore, in order to facilitate dogs’ attachment style recognition and improve inter-observer agreement, we suggest that dogs’ attachment style classification give more weight to those key concepts that characterize the evolution of attachment behavioral patterns throughout the SSP rather than focusing on specific behaviors or behavioral sequences within each episode.

## Figures and Tables

**Table 1:** Behaviors observed during SSP in protocol I: coded behavior, episode in which it was analyzed, brief description, and variable measured (D = duration). All behaviors were measured in seconds.

Behavior	Episodes	Brief Description	Variable
Proximity to/Contact with Owner Stranger	4.I, 7.I 3.I, 6.I	Being close to (in the range of one dog body-length) or in physical contact with the person	D
Approach Owner Stranger	4.I, 7.I 3.I, 6.I	Approaching the person while clearly visually oriented to the person. Approach is not recorded if the dog stops.	D
Visual orientation to Owner Stranger Door	4.I, 7.I 3.I, 6.I 3.I, 4.I, 5.I, 6.I, 7.I	Staring fixedly. In case of a person the behavior may not be reciprocated	D
Avoidance of the owner	4.I, 7.I	Every action aimed to avoid interaction with the person, such as turning the head away from the person and/or increasing physical distance by moving away.	D
Exploration	3.I, 4.I, 5.I, 6.I, 7.I	Activity directed toward physical aspects of the environment, including sniffing, close visual inspection, distal visual inspection, and gentle oral examination such as licking	D
Stress	3.I, 4.I, 5.I, 6.I, 7.I	Lip-licking, yawning, shaking, self-scratching, self-grooming, escape attempts, hypersalivation	D
Proximity to door	3.I, 4.I, 5.I, 6.I, 7.I	Standing close to the door (<1 m) regardless of whether the face was oriented to the exit	D
Behaviors toward the door	3.I, 4.I, 5.I, 6.I, 7.I	All active behaviors resulting in physical contact with the door, including scratching the door with the paws, jumping on the door, pulling on the door handle with the forelegs or mouth	D
Vocalization	3.I, 4.I, 5.I, 6.I, 7.I	Whining, yelping, barking	D

**Table 2:** Dog attachment styles and relative descriptions (modified from Somolon et al. [20]).

Attachment Style	Description (Current Study)	Description (Solomon et al. [20])
Secure	The dog actively seeks proximity with the caregiver (e.g., approach, physical proximity, contact and/or persistent gazing), and such proximity is increased in quality and/or intensity after separation, especially upon the second reunion. The reunion with the caregivers (their mere presence or, for other dogs, the proximity or the contact with them) is responsible for calming the dog in case he was distressed at separation. It may take some time, but during the reunion episode the dog, if excited/distressed at the beginning of the episode itself, appears reassured by the presence of or interaction with the caregiver, so that he can either explore the environment, lay down, remain in proximity/contact with the caregiver, always in a relaxed manner. The dog may show some interest in the stranger, thus can greet her and stay in proximity/contact with her. However, the dog shows a preference for the caregiver (e.g., more intense greeting, longer or deeper proximity seeking, reassurance). During separation, especially when left alone in the room, the dog may show some search of the caregiver (e.g., sniffing as searching, going close to the chair of the caregiver, looking at the door, staying close to the door, scratching at the door), and may display some distress at separation (e.g., vocalizations). When separated from the caregiver but in the presence of the stranger, the dog may remain close to the stranger for reassurance. Before separation, especially before the first separation, the dog may show interest in the environment, thus exploring the room and the stranger (for the latter, it can be displayed as sniffing, greeting). Such interest for the environment may remain even after separation, but it is usually overshadowed by the increase in proximity seeking. In certain cases, such as dogs not used to novel environments or places similar to the experimental room, exploration may be reduced.	Dog shows active proximity and contact seeking, i.e. approaches caregiver promptly at reunion and makes physical contact or signals for contact. Once contact is achieved, the dog does not break contact for at least 10 s. There is little or no gaze aversion or proximity avoidance; there is little or no resistance to contact or interaction. In the pre-separation episodes the dog engages in some independent or social play or exploration. (Unlike human infants, many dogs showed little or no independent exploration or play by the 2nd reunion). Sleeping or lying down after proximity or physical contact was sought and achieved at reunion did not disqualify a dog from placement in the secure category unless it was associated with ignoring the overtures or requests of the caregiver. The dog shows some active search, but not necessarily distress, in all separations.
Insecure-avoidant	The dog shows little or no tendency to actively seek proximity with the caregiver (e.g., approach, physical contact and/or persistent gazing), and such proximity is not increased in quality and/or intensity after separation, instead it may be reduced upon the second reunion. The dog shows some proximity avoidance: the dog may follow or approach the caregiver but the sequence is quickly interrupted, so that the dog turns, looks or moves away. The dog shows some gaze aversion, e.g., alternate gazing from the caregiver to somewhere else not clearly identified (i.e., not the door). The dog may show some interest in the stranger, thus can greet her and stay in proximity/contact with her. The dog does not show a marked preference for the caregiver (e.g., more intense greeting, longer or deeper proximity seeking, reassurance). During separation, the dog may display some/little search of the caregiver (e.g., sniffing as searching, going close to the chair of the caregiver, looking at the door, staying close to the door), and shows little distress at separation (e.g., vocalizations). Distress at separation may be more pronounced when the dog is left completely alone. When separated from the caregiver but in the presence of the stranger, the dog may remain close to the stranger for reassurance. Before separation, especially before the first separation, the dog may show interest in the environment, thus exploring the room and the stranger (for the latter, it can be shown as sniffing, greeting). Such interest for the environment may remain even during and after separation. In certain cases, such as dogs not used to novel environments or places similar to the experimental room, exploration may be reduced.	Dog shows little tendency to approach, to seek contact, or to follow. Dog turns, looks, or moves away and/or shows lack of response to invitations to approach or interact for the first 30 s of reunion or more. Dog explores the room and objects during pre- and post-separation. There is little active search for caregiver during separations, except when the dog is left alone in the room.
Insecure-ambivalent	The dog actively and obviously seeks proximity with the caregiver (e.g., physical proximity, contact and/or persistent gazing), and such proximity is increased in quality and/or intensity after separation, especially upon the second reunion. Differently from the secure style, the dog cannot find reassurance in the caregiver, so that the dog makes strong efforts to maintain physical contact with the caregiver (e.g., physically intrusive behavior) and this is combined with persistent distress (e.g., the dog can keep vocalizing, bite/chew the owner). The dog may show interest in the stranger, thus can greet her and stay in proximity/contact with her. However, the dog shows a marked preference for the caregiver (e.g., more intense greeting, longer or deeper proximity seeking, reassurance). During separation, especially when left alone in the room, the dog shows some search of the caregiver (e.g., sniffing as searching, going close to the chair of the caregiver, looking at the door, staying close to the door, scratching at the door), and displays evident distress at separation (e.g., frequent vocalizations, pacing). When separated from the caregiver but in the presence of the stranger, the dog may remain close to the stranger for reassurance. Before separation, especially before the first separation, the dog may show little interest in the environment, not exploring the room nor focusing on the stranger (e.g., sniffing, greeting) for a long time. If displayed, such interest for the environment does not remain after separation.	On reunion, the dog makes strong efforts to maintain physical contact mixed with persistent distress and/or physically intrusive behavior directed toward the caregiver. The dyad is characterized by a degree of conflict regarding physical contact or play activities (e.g. the dog attempts to maintain contact and is uncooperative with the caregiver’s attempt to encourage play or exploration; or, once proximity is sought by the dog, caregiver actively maintains contact despite the dog’s signals of readiness to explore). In the pre-separation episodes, the dog shows little interest in exploration and/or the playmate and clearly prefers to remain nearby the caregiver. During separations, the dog makes frequent distress vocalizations and shows some active search (though he/she may also remain near the playmate for reassurance).
Disorganized	There is not a clear pattern; behavior is inexplicable or contradictory in the context of interaction with the caregiver, and this lack of organization has to be frequent, extreme, or extensive and more evident in the caregiver’s presence than absence. Particular relevance is given to repeated manifestations of disorganization, the appearance of several different indices of disorganization, and disorganized behavior displayed immediately after reunion.	Disorganized behavior refers to behavior that is inexplicable or contradictory in the context of interaction with an attachment figure and/or with respect to the organized A, B, or C patterns of attachment. It often manifests as a sudden and marked disruption of ongoing proximity seeking, contact maintaining, avoidance, or contact resistance; if strongly present, disorganized behaviors can make it difficult to perceive any underlying classification.
Unclassified	The dog behavior seems disturbed but too ambiguous to classify, and an alternative underlying condition can be supposed, either a physical illness or a behavioral disorder. For instance, the dog shows constantly repetitive behaviors regardless of the caregiver presence (possibly due to neurological or compulsive disorder), or the dog shows lethargy.	The dog’s behavior seems disturbed but it is too ambiguous to classify. For example, it is unclear whether the dog is frequently dissociating in the caregiver’s presence or simply reacting to distant sounds that the coder cannot hear; or, the dog is skittish and circles the room repeatedly, whether or not the caregiver is present, suggesting a neurological or compulsive condition; or, the dog’s greetings and approaches to the caregiver are markedly lethargic, possibly suggesting depression or physical illness.

**Table 3:** Behaviors observed during SSP in protocol II: coded behavior, episodes in which it was analyzed, brief description, and variables measured (F = frequency, D = duration).

Behavior	Episodes	Brief Description	Variable
Looking at owner stranger door	3.II, 4.II, 7.II 2.II, 5.II, 7.II 2.II, 4.II, 6.II	Visually oriented to the person/door	D
Proximity to/Contact with owner stranger both owner and stranger	3.II, 4.II, 7.II 2.II, 5.II, 7.II 7.II	Within one body-length from the person or in contact with person. Contact was recorded only if it was actively initiated by the dog.	D
Proximity to the door	2.II, 4.II, 6.II	Within one body-length from the door	D
Vocalizations	2.II, 3.II, 4.II, 5.II, 6.II, 7.II	Whining, yelping and barking	D
Individual play	2.II, 3.II, 4.II, 5.II, 6.II, 7.II	Any vigorous or galloping gaited behavior directed toward a toy when clearly not interacting with any participants; including chewing, biting, shaking, scratching or batting with the paw, chasing rolling balls and tossing using the mouth	D
Exploration of environment	2.II, 4.II, 6.II, 7.II	Sniffing the physical environment, regardless from movement	D
Stress	2.II, 3.II, 4.II, 5.II, 6.II, 7.II	Lip-licking, head-turning, yawning, shaking, self-scratching, self-grooming	F
Greeting interruption	3.II, 7.II	The dog actively interrupts greeting the owner for at least three seconds while focusing on something else (e.g., toys, other person, environment)	F
Behaviors against the door	2.II, 4.II, 6.II	All active behaviors resulting in physical contact with the door, including scratching the door with the paws, jumping on the door, pulling on the door handle with the forelegs or mouth	D

**Table 4:** Behavioral scores for protocol II, episodes in which they were measured and relative scoring system.

Behavior	Episode	Scoring
Social play behavior with owner stranger	3.II, 4.II 2.II, 5.II	+3: dog responds to the person’s first attempt to engage +2: dog responds to the person’s second attempt to engage +1: dog responds to the person’s third attempt to engage 0: dog does not respond to the person attempts to engage
Greeting behavior toward owner stranger	3.II, 7.II 5.II, 7.II	+2: full approach with physical contact +1: approach initiation 0: neutral −1: any sign of avoidance behavior

**Table 5:** Comparison of secure and avoidant dogs’ behavior during SSP (Protocol I).

Episode	Category	Behavior	Min–Max (Median) for Secure Dogs	Min–Max (Median) for Avoidant Dogs	Results	Summary
3.I	Protest at separation	Oriented to door/window	14–102 (58.00)	38–111 (99.00)	U = 22.50, *p* = 0.036	Sec < Av ^*^
	Secure base	Exploration	7–90 (29.50)	0–79 (6.00)	U = 21.50, *p* = 0.030	Sec > Av ^*^
4.I	Proximity seeking	Oriented to owner	0–66 (23.50)	1–28 (7.50)	U = 26.50, *p* = 0.066	**Sec > Av**
	Protest at separation	Vocalizations	0–17 (0.00)	0–18 (4.00)	U = 32.00, *p* = 0.092	Sec < Av
	Stress	Stress	0–45 (10.00)	3–8 (4.00)	U = 26.50, *p* = 0.065	Sec > Av
5.I	Stress	Stress	0–4 (0.50)	0–7 (2.50)	U = 27.50, *p* = 0.064	Sec < Av
6.I	Proximity seeking	Proximity to/Contact with stranger	10–116 (65.50)	0–91 (4.00)	U = 14.00, *p* = 0.008	Sec > Av ^*^
7.I	Proximity seeking	Proximity to/Contact with owner	13–120 (113.50)	0–118 (18.50)	U = 21.00, *p* = 0.027	Sec > Av ^*^
	Protest at separation	Vocalizations	0–12 (0.00)	0–39 (3.50)	U = 21.00, *p* = 0.011	Sec < Av ^*^
		Oriented to door/window	0–62 (13.00)	11–92 (41.00)	U = 22.50, *p* = 0.036	Sec < Av ^*^
	Stress	Stress	3–44 (7.50)	0–9 (3.50)	U = 25.00, *p* = 0.052	Sec > Av

In bold = behavioral results matching hypothesis (for hypotheses see Table S3), ^*^ = *p* < 0.05.

**Table 6:** Pair-wise comparison of secure dogs’ behavior between episodes (Protocol I). Lower = the episode that in the SSP precedes the other to which it is compared, i.e., the higher.

Episode	Category	Behavior	Min–Max (Median) Lower Episode	Min–Max (Median) Higher Episode	Results	Summary
3.I vs. 4.I (1st separation from owner vs. 1st separation from stranger)	Protest at separation	Vocalizations	0–67 (2.00)	0–17 (0.00)	Z = −2.402, *p* = 0.016	3.I > 4.I ^*^
		Proximity to door	0–120 (17.50)	0–104 (0.00)	Z = −2.457, *p* = 0.014	3.I > 4.I ^*^
		Oriented to door	14–102 (58)	0–98 (19)	Z = −3.845, *p* < 0.001	3.I > 4.I ^*^
	Proximity seeking	Proximity to/Contact with stranger vs. proximity to/contact with owner	0–87 (20)	4–120 (58.50)	Z = −2.940, *p* = 0.003	3.I < 4.I ^*^
		Oriented to stranger vs. oriented to owner	0–34 (6)	0–66 (23.50)	Z = −2.593, *p*=0.010	3.I < 4.I ^*^
	Stress	Stress	0–45 (2.50)	0–45 (10.00)	Z = −3.184, *p* = 0.001	3.I < 4.I ^*^
3.I vs. 5.I (1st separation from owner vs. 2nd separation from owner and stranger –alone)	Protest at separation	Proximity to door	0–120 (17.50)	0–120 (76.00)	Z = −2.510, *p* = 0.012	3.I < 5.I ^*^
		Oriented to door	14–102 (58)	23–110 (80.5)	Z = −1.939, *p* = 0.053	**3.I < 5.I**
	Stress	Stress	0–45 (2.5)	0–4 (0.5)	Z = −2.211, *p* = 0.027	3.I > 5.I ^*^
3.I vs. 6.I (1st separation from owner vs. 2nd separation from owner)	Protest at separation	Vocalizations	0–67 (2.00)	0–33 (2.80)	Z = 2.552, *p* = 0.011	3.I > 6.I ^*^
		Proximity to door	0–120 (17.50)	0–89 (8.00)	Z = −2.045, *p* = 0.041	3.I > 6.I ^*^
	Proximity/Contact seeking	Proximity to/Contact with stranger	0–87 (20.00)	10–116 (65.50)	Z = −3681, *p* < 0.001	3.I < 6.I ^*^
		Oriented to stranger	0–34 (6.00)	0–48 (11.00)	Z = −1.889, *p* = 0.059	**3.I < 6.I**
4.I vs. 7.I (1st reunion with owner vs. 2nd reunion with owner)	Proximity seeking	Proximity to/Contact with owner	4–120 (58.50)	13–120 (113.50)	Z = −2.642, *p* = 0.008	4.I < 7.I ^*^
6.I vs. 7.I (2nd separation from owner vs. 2nd separation from stranger)	Protest at separation	Proximity to door	0–89 (8.00)	0–59 (0.00)	Z = −2.937, *p* = 0.003	6.I > 7.I ^*^
		Oriented to door	3–99 (55.50)	0–62 (13.00)	Z = −3.660, *p* < 0.001	6.I > 7.I ^*^
		Vocalizations	0–33 (1.50)	0–12 (0.00)	Z = −1.917, *p* = 0.055	**6.I > 7.I**
	Proximity seeking	Approach stranger vs. approach owner	0–8 (0.00)	0–3 (0.00)	Z = −1.875, *p* = 0.061	6.I > 7.I
		Proximity to/Contact with stranger vs. proximity to/Contact with owner	10–116 (65.50)	13–120 (113.50)	Z = −2.418, *p* = 0.016	6.I < 7.I ^*^
	Stress	Stress	0–12 (3.00)	3–44 (7.50)	Z = −2.989, *p* = 0.003	6.I < 7.I ^*^

In bold = behavioral results matching hypothesis (for hypotheses see Table S3), ^*^ = *p* < 0.05.

**Table 7:** Pair-wise comparison of avoidant dogs’ behavior between episodes (Protocol I). Lower = the episode that in the SSP precedes the other to which it is compared, i.e., the higher.

Episode	Category	Behavior	Min–Max (Median) Lower Episode	Min–Max (Median) Higher Episode	Results	Summary
3.I vs. 4.I (1st separation from owner vs. 1st separation from stranger)	Protest at separation	Oriented to door/window	38–111 (99.00)	2–43 (16.00)	Z = −2.201, *p* = 0.028	3.I > 4.I ^*^
		Proximity to door	5–106 (57.00)	0–15 (0.50)	Z = −2.201, *p* = 0.028	3.I > 4.I ^*^
	Secure base	Exploration	0–79 (6.00)	0–95 (49.00)	Z = −1.992, *p* = 0.046	3.I < 4.I ^*^

In bold = behavioral results matching hypothesis (for hypotheses see Table S3), ^*^ = *p* < 0.05.

**Table 8:** Comparison of secure and avoidant dogs’ behavior during SSP (Protocol II).

Episode	Category	Behavior (Variable)	Min–Max (Median) for Secure Dogs	Min–Max (Median) for Avoidant Dogs	Results	Summary
2.II	Proximity seeking	Proximity to/Contact with stranger	0–60253 (2870.50)	8749–59755 (38754.50)	U = 48.00, *p* = 0.049	Sec < Av ^*^
3.II	Proximity seeking	Greeting score toward owner	0–2 (1.50)	0–2 (0.50)	U = 50.00, *p* = 0.046	Sec > Av ^*^
4.II	Protest at separation	Vocalizations	0–10751 (0)	0–21257 (376.00)	U = 52.00, *p* = 0.018	Sec < Av ^*^
	Stress	Stress	0–7 (1.00)	1–4 (2.00)	U = 52.00, *p* = 0.095	Sec < Av
6.II	Protest at separation	Vocalizations	0–32506 (1996.50)	1168–40740 (7374.00)	U = 55.00, *p* = 0.097	Sec < Av
7.II	Proximity seeking	Greeting score toward owner	−1–2 (2.00)	−1–2 (−0.50)	U = 35.00, *p* = 0.007	Sec > Av ^*^

In bold = behavioral results matching hypothesis (for hypothesis see Table S3), ^*^ = *p* < 0.05.

**Table 9:** Pair-wise comparison of secure dogs’ behavior between episodes (Protocol II). Lower = the episode that in the SSP precedes the other to which it is compared, i.e., the higher.

Episode	Category	Behavior	Min–Max (Median) Lower Episode	Min–Max (Median) Higher Episode	Results	Summary
2.II vs. 4.II (1st separation from owner vs. 1st separation from stranger)	Proximity seeking	Proximity to/Contact with stranger vs. owner	0–60253 (4000.00)	0–60500 (37749.50)	Z = −2.116, *p* = 0.034	2.II < 4.II ^*^
		Looking at stranger vs. owner	0–13744 (3747.00)	0–35000 (7997.50)	Z = −2.931, *p* = 0.003	2.II < 4.II ^*^
	Secure base	Play score stranger vs. owner	0–3 (0)	0–3 (3)	Z = 3.633, *p* < 0.001	2.II < 4.II ^*^
2.II vs. 5.II (owner absence vs. owner presence)	Secure base	Play score stranger vs. stranger	0–3 (0)	0–3 (2)	Z = −2.347, *p* = 0.019	2.II < 5.II ^*^
2.II vs. 6.II (1st separation from owner vs. 2nd separation from owner and stranger-alone)	Protest at separation	Vocalizations	0–15754 (0)	0–32506 (1996.50)	Z = −3.003, *p* = 0.003	2.II < 6.II ^*^
		Looking at door	0–57253 (38 256.00)	9999–57516 (43761.00)	Z = −2.077, *p* = 0.038	2.II < 6.II ^*^
3.II vs. 5.II (1st reunion with owner vs. 1st reunion with stranger)	Secure base	Play score owner vs. stranger	0–3 (3)	0–3 (2)	Z = −3.126, *p* = 0.002	3.II > 5.II ^*^
	Proximity seeking	Greeting score owner vs. stranger	1–2 (1.5)	0–1 (1)	Z = −3.373, *p* = 0.001	3.II > 5.II ^*^
		Proximity to/Contact with owner vs. stranger	3499–48495 (33121.50)	0–43753 (7867.50)	Z = −3.758, *p* < 0.001	3.II > 5.II ^*^
		Looking at owner vs. stranger	0–41755 (13124)	0–37506 (8899.50)	Z = −2.150, *p* = 0.032	3.II > 5.II ^*^
3.II vs. 7.II (1st reunion with owner vs. 2nd reunion with owner and stranger)	Proximity seeking (reverse)	Greeting interruption with owner	0–5 (1.50)	0–3 (1.00)	Z = −2.847, *p* = 0.004	3.II > 7.II ^*^
7.II vs. 7.II (2nd reunion with owner vs. 2nd reunion with stranger)	Proximity seeking	Proximity to/Contact with owner vs. stranger	0–59004 (20626.50)	0–17498 (0)	Z = −3.610, *p* < 0.001	Owner > stranger ^*^
		Looking at owner vs. stranger	0–23746 (3123.00)	0–21752 (0)	Z = −3.391, *p* = 0.001	Owner > stranger ^*^
		Greeting score owner vs. stranger	0–2 (2)	−1–3 (1)	Z = −3.108, *p* = 0.002	Owner > stranger^*^

In bold = behavioral results matching hypothesis (for hypothesis see Table S3), ^*^ = *p* < 0.05.

**Table 10:** Pair-wise comparison of avoidant dogs’ behavior between episodes (Protocol II). Lower = the episode that in the SSP precedes the other to which it is compared, i.e., the higher.

Episode	Category	Behavior	Min–Max (Median) Lower Episode	Min–Max (Median) Higher Episode	Results	Summary
2.II vs. 4.II (1st separation from owner vs. 1st separation from stranger)	Stress	Stress	0–2 (0.00)	1–4 (2.00)	Z = −2.041, *p* = 0.041	2.II < 4.II ^*^
2.II vs. 6.II (1st separation from owner vs. 2nd separation from owner and stranger-alone)	Protest at separation	Vocalizations	0–24490 (1377.00)	1168–40740 (7374.00)	Z = −1.992, *p* = 0.046	2.II < 6.II ^*^
		Looking at door	6250–45999 (24128.50)	29998–56497 (44376.50)	Z = −2.201, *p* = 0.028	2.II < 6.II ^*^

In bold = behavioral results matching hypothesis (for hypotheses see Table S3), ^*^ = *p* < 0.05.

## Data Availability

The data presented in this study are available on request from the corresponding author.

## Supplementary Materials

The following are available online at https://www.mdpi.com/2076-2615/11/1/14/s1, Table S1 Demographic details for subjects in protocol I and II, Table S2 Behaviors analysed and corresponding episodes for which comparisons were made, Table S3 Hypotheses of expected results for behavioral comparison between and within secure and avoidant dogs.
