# Supplementary material for: Quantitative Behavioral Analysis and Qualitative Classification of Attachment Styles in Domestic Dogs: Are Dogs with a Secure and an Insecure-Avoidant Attachment Different?
Source: Animals (Basel). 2020 Dec 23;11(1):14. doi: 10.3390/ani11010014 (PMC7823664; doi:10.3390/ani11010014)
Supplement: Supplementary file 1 [file animals-11-00014-s001.pdf]

animals

MDPI

Article

Quantitative Behavioral Analysis and Qualitative Classification of Attachment Styles in Domestic Dogs: Are Dogs with a Secure and an Insecure-Avoidant Attachment Different?

Giacomo Riggio, Angelo Gazzano, Borbála Zsilák, Beatrice Carlone and Chiara Mariti

Table S1. Demographic details for subjects in protocol I and II (f = female, m = male, i = intact, s = spayed, c = castrated).

Demographic details for subjects in protocol I				
**Number**	**Dog's Breed**	**Dog' Sex**	**Dog's Age (Months)**	**Owner's Gender**
1	German Shepherd	f,i	60	f
2	Mix-breed	m,c	84	m
3	Welsh Corgi	f,i	42	f
4	Mix-breed	f,s	240	f
5	Labrador Retriever	f,i	34	f
6	Border Collie	f,i	48	f
7	Border Collie	f,i	38	f
8	German Shepherd	f,i	28	f
9	Labrador Retriever	m,i	68	m
10	Welsh Corgi	m,i	42	f
11	Poodle	m,i	30	f
12	Collie	f,s	36	f
13	Springer Spaniel	m	60	m
14	Labrador Retriever	m	48	m
15	Border Collie	f,s	48	f
16	Mix-breed	m	48	f
17	Labrador Retriever	f,i	47	f
18	Bearded Collie	f,i	28	f
19	Bernese Mountain Dog	f,i	42	m
20	Belgian Tervuren	f,i	24	f
21	Golden Retriever	f,i	84	m
22	Border Collie	m,i	48	f
23	Australian Kelfie	f,i	36	f
24	Mix-breed	m,c	48	m
25	Mix-breed	f,s	32	f
26	Beagle	m,i	73	f
27	Mix-breed	f,s	36	f
**Demographic details for subjects in protocol II**				
**Number**	**Dog's Breed**	**Dog' Sex**	**Dog's Age (Months)**	**Owner's Gender**
1	Labrador Retriever	m,i	18	m
2	German Shepherd	m,i	96	m
3	German Shepherd	m,i	60	f
4	German Shepherd	m,i	84	f
5	Mix-breed	f,s	16	f
6	Mix-breed	m,i	78	m
7	German Shepherd	f,s	120	m

8	Beagle	m,i	72	m
9	Labrador Retriever	f,i	36	m
10	Labrador Retriever	f,i	36	m
11	Belgian Malinois	m,i	26	f
12	Labrador Retriever	f,s	48	m
13	Labrador Retriever	m,i	14	m
14	Labrador Retriever	m,i	42	m
15	Border Collie	f,i	27	m
16	Springer Spaniel	m,i	24	f
17	Labrador Retriever	m,c	15	f
18	Labrador Retriever	m,i	16	m
19	Labrador Retriever	f,s	84	f
20	Irish Setter	f,i	24	f
21	Belgian Malinois	m,i	12	f
22	Border Collie	m,i	24	m
23	Labrador Retriever	f,i	14	m
24	Mix-breed	m,i	42	m
25	German Shepherd	f,s	96	f
26	Golden Retriever	m,i	84	f
27	German Shepherd	f,i	96	f
28	Labrador Retriever	f,i	15	m
29	Flat-Coated Retriever	f,i	15	f
30	Border Collie	f,i	24	f
31	Mix-breed	m,c	30	f
32	Mix-breed	m,i	36	m
33	Mix-breed	m,c	18	f
34	Mix-breed	f,s	48	m
35	Mix-breed	f,i	14	f
36	Labrador Retriever	f,i	29	f
37	Labrador Retriever	m,i	48	f
38	Pit Bull	f,i	21	f
39	Dobermann	m,i	84	m
40	Jack Russell Terrier	f,i	29	f

Table S2. Behaviors analysed and corresponding episodes for which comparisons were made.

**Protocol I**	
**Behavior**	**Episodes**
Proximity to/Contact with	
Stranger vs owner	3 vs 4, 6 vs 7
Stranger vs stranger	3 vs 6
Owner vs owner	4 vs 7
Approach	
Stranger vs owner	3 vs 4, 6 vs 7
Stranger vs stranger	3 vs 6
Owner vs owner	4 vs 7
Visual orientation to	
Owner vs owner	4 vs 7
Stranger vs owner	3 vs 4
Stranger vs stranger	3 vs 6
Door	3 vs 4, 3 vs 5, 6 vs 7
Avoidance of the owner	4 vs 7

Exploration	3 vs 4, 6 vs 7
Stress	3 vs 4, 6 vs 7
Proximity to door	3 vs 4, 3 vs 5, 3 vs 6, 6 vs 7
Behaviors towards the door	3 vs 4, 3 vs 5, 3 vs 6, 6 vs 7
Vocalization	3 vs 4, 3 vs 5, 3 vs 6, 6 vs 7
**Protocol II**	
**Behavior**	**Episodes**
Looking at<br>Owner vs stranger<br>Owner vs owner<br>Door	 2 vs 4 , 3 vs 5, 7 vs 7<br>3 vs 7<br>2 vs 6
Proximity to/Contact with<br>Owner vs stranger<br>Owner vs owner	 2 vs 4 , 3 vs 5, 7 vs 7<br>3 vs 7
Proximity to the door	2 vs 6
Ears back	3 vs 5, 3 vs 7, 5 vs 7
Vocalizations	2 vs 6
Individual play	2 vs 4, 2 vs 6
Exploration of environment	2 vs 4, 2 vs 6
Stress	2 vs 4, 2 vs 6
Greeting interruption<br>Owner vs stranger<br>Owner vs owner<br>Stranger vs stranger	 3 vs 5<br>3 vs 7<br>5 vs 7
Behaviors against the door	2 vs 6
Social play (score)<br>Owner vs stranger<br>Owner vs owner	 2 vs 3, 2 vs 4, 3 vs 5<br>2 vs 5, 3 vs 4
Greeting (score)<br>Owner vs stranger<br>Owner vs owner<br>Stranger vs stranger	3 vs 5, 7 vs 7<br>3 vs 7<br>5 vs 7

Table S3. Hypotheses of expected results for behavioral comparison between and within secure and avoidant dogs.

**Hypotheses of behavioral differences between secure and avoidant dogs in protocol I**	
**Episode**	**Hypothesis**
3.I	Protest at separation: Secure>Avoidant<br>Stress: Secure>Avoidant
4.I	Proximity seeking: Secure>Avoidant
5.I	Protest at separation: Secure>Avoidant<br>Stress: Secure>Avoidant
6.I	Proximity seeking: Secure>Avoidant
7.I	Proximity seeking: Secure>Avoidant
**Hypotheses of behavioral differences between episodes in secure dogs in protocol I**	
**Episode**	**Hypothesis**
3.I vs 4.I	Protest at separation: 3.I>4.I<br>Proximity seeking: 3.I<4.I

(1st separation from owner vs 1st separation from stranger)	Stress: 3.I>4.I
3.I vs 5.I (1st separation from owner vs 2nd separation from owner and stranger –alone)	Protest at separation: 3.I<5.I<br>Stress:3.I<5.I
3.I vs 6.I (1st separation owner vs 2nd separation owner)	Protest at separation: 3.I<6.I<br>Proximity seeking: 3.I<6.I
4.I vs 7.I (1st reunion with owner vs 2nd reunion with owner)	Proximity seeking: 4.I<7.I
6.I vs 7.I (2nd separation from owner vs 2nd separation from stranger)	Protest at separation: 6.I>7.I<br>Proximity seeking: 6.I<7.I<br>Stress: 6.I>7.I
**Hypotheses of behavioral differences between episodes in avoidant dogs in protocol I**	
**Episode**	**Hypothesis**
3.I vs 4.I (1st separation from owner vs 1st separation from stranger)	Any behavioral categories: 3.I≈4.I
**Hypotheses of behavioral differences between secure and avoidant dogs in protocol II**	
**Episode**	**Hypothesis**
2.II	Proximity seeking: Sec>Av
3.II	Proximity seeking: Sec>Av
4.II	Proximity seeking: Sec>Av
6.II	Protest at separation: Sec>Av
7.II	Proximity seeking: Sec>Av
**Hypotheses of behavioral differences between episodes in secure dogs in protocol II**	
**Episode**	**Hypothesis**
2.II vs 3.II (1st separation from owner vs 1st re-union with owner)	Secure base:<br>2.II<3.II
2.II vs 4.II (1st separation from owner vs 1st sep-aration from stranger)	Proximity seeking: 2.II<4.II
	Secure base: 2.II<4.II
2.II vs 5.II (owner absence vs owner presence)	Secure base: 2.II<5.II
2.II vs 6.II (1st separation from owner vs 2nd separation from owner and stranger - alone)	Protest at separation: 2.II<6.II
3.II vs 5.II (1st reunion with owner vs 1st reunion with stranger)	Secure base: 3.II>5.II
	Proximity seeking:<br>3.II>5.II
3.II vs 7.II (1st reunion with owner vs 2nd reun-ion with owner and stranger)	Proximity seeking:<br>3.II<7.II
7.II vs 7.II (2nd reunion with owner vs 2nd reunion with stranger)	Proximity seeking:<br>Owner>stranger
**Hypotheses of behavioral differences between episodes in avoidant dogs in protocol II**	
**Episode**	**Hypothesis**
2.II vs 4.II (1st separation from owner vs 1st separation from stranger)	Stress: 2≈4
